# Blood Profiling of Captive and Semi-Wild False Gharial in Peninsular Malaysia

**DOI:** 10.3390/ani11061481

**Published:** 2021-05-21

**Authors:** Mohd Qayyum Ab Latip, Tengku Rinalfi Putra Tengku Azizan, Hafandi Ahmad, Hasliza Abu Hassim, Mohd Hezmee Mohd Noor, Muhammed Mikail

**Affiliations:** 1 Department of Veterinary Preclinical Sciences, Faculty of Veterinary Medicine, Universiti Putra Malaysia, Serdang 43400, Selangor Darul-Ehsan, Malaysia; qayyum2188@gmail.com (M.Q.A.L.); hafandi@upm.edu.my (H.A.); haslizaabu@upm.edu.my (H.A.H.); hezmee@upm.edu.my (M.H.M.N.); ningimikail74@gmail.com (M.M.); 2 Wildlife Research Centre, Faculty of Veterinary Medicine, Universiti Putra Malaysia, Serdang 43400, Selangor Darul-Ehsan, Malaysia; 3 Laboratory of Sustainable Animal Production and Biodiversity, Institute of Tropical Agriculture and Food Security (ITAFoS), Universiti Putra Malaysia, Serdang 43400, Selangor Darul-Ehsan, Malaysia; 4 University Agriculture Park, Universiti Putra Malaysia, Serdang 43400, Selangor Darul-Ehsan, Malaysia

**Keywords:** false gharial, hematology, serum biochemistry, zoo, conservation center

## Abstract

**Simple Summary:**

Ill or injured wildlife species are been rescued, treated and or rehabilitated usually at the wildlife rescued centers, zoos clinic facilities and or wildlife conservation centers. The false gharial also known as Malayan gharial is a crocodile species native to Peninsular Malaysia, Borneo and Indonesia with extirpation in Singapore, Vietnam and Thailand. The false gharial is facing a threat of extinction due to habitat destruction and hunting Policies were implemented to conserve this species through the establishment of Ex-Situ Conservation centers, as such to maintain the health and welfare status of this species while in captivity, understanding its normal Haematology and serum biochemistry values becomes necessary to save the false gharial from extinction.

**Abstract:**

The involvement of veterinary medicine in wildlife research has played an important role in understanding the health status of various wildlife species. Health status is a very important aspect of species conservation. However, it requires a widely employed knowledge of veterinary clinical pathology, as a diagnostic tool in diagnosing the various disease conditions of wildlife species. Notwithstanding, a gap exists in the literature about the clinical pathology of the false gharial, due to the lack of normal reference values for hematological and serum biochemical analysis. The present study investigated the normal blood profile of 10 healthy false gharials, from two different zoos, and wildlife conservation centers located in three different states of Peninsular Malaysia. Blood samples were collected from the lateral caudal vein and divided into a vacutainer without anticoagulant for biochemical analysis, and a lithium heparin vacutainer (containing sodium heparin) for hematological studies. The results of the study indicated that the false gharial has a smaller erythrocyte dimension compared to other crocodilian species. At the same time the study revealed that the false gharial in a natural captive pond showed more leukocytes than false gharial kept in zoos, hence, habitat and environmental factors significantly affect hematological values. The biochemistry values also showed differences between the false gharial in different environmental conditions. Total protein, albumin (Alb), globulin (Glob), and Alb: Glob ratio were higher in false gharials kept in wildlife conservation centers than in false gharials kept in zoos. The values obtained in this study provide baseline data of hematological and serum biochemical values of the false gharial for future research and routine clinical diagnosis.

## 1. Introduction

The false gharial (*Tomistoma schlegelii)* is a freshwater crocodilian with a distinctively narrow and long snout, and endemic to Indonesia and Malaysia [1,2]. False gharial distribution is limited to Indonesia (Kalimantan, West Java, and East Sumatra), Malaysia (Peninsular Malaysia, Sarawak), and Brunei, with no definitive population estimate for the false gharial [3].

These species were reported to be extinct in Thailand where the last sightings were made in 1970 [3]. To date, research about the false gharial, in both Malaysia and Indonesia, has focused on the census of false gharial in its natural habitats and other issues related to the conservation of its habitats [1,2,3,4,5,6].

The false gharial conservation status is vulnerable, as classified by the International Union for Conservation of Nature (IUCN) Red List [7], and it is described as having high research and conservation priority by the IUCN Crocodile Specialist Group (CSG) [8,9]. However, in Peninsular Malaysia, the false gharial is protected under Wildlife Conservation Act 2010, and it is listed as a protected animal in the State of Sarawak Wild Life Protection Ordinance (1998); prohibiting its hunting, killing, or the selling of wild false gharial in the state [10].

Clinical pathology is used to monitor the health status of various animal species, including false gharial. However, the living conditions of the false gharial in the wild have not been adequately reported, because they are very elusive, and therefore knowledge on how to emulate the natural environment for the captive false gharial is lacking. Accordingly, a gap exists in the knowledge, about the health status of captive false gharial in Peninsular Malaysia due to lack of adequate research on the species.

Knowledge about the haematological and serum biochemistry values of false gharial and other reptiles has been used as an aid or guide in the detection and diagnosis of various hemoparasites, and other infectious diseases, such as chlamydiosis and poxvirus infection [11,12]. The haematology and serum biochemical values in false gharial were preliminary investigated by [13] in a captive individual in Thailand, where parameters such as the haematocrit, haemoglobin concentration, erythrocytes count, leukocytes count, thrombocyte count, red cell indices, and 17 serum biochemistry parameters were analysed, with significant differences between male and female group in detected only in and the red cell indices (MCHC).

The aim of this study was to draw up a complete haematological and serum biochemical profile of Malaysian false gharial and to compare the profile of individuals kept in captive and semi-wild conditions in different states of Peninsular Malaysia, which subsequently will aid the interpretation of laboratory results. In this present study, we hypothesized the occurrence of differences in some blood parameters in relation to different habitat conditions.

## 2. Materials and Methods

### 2.1. False Gharial and Blood Sampling

#### 2.1.1. Study Site, Ethical Approval, and Animals

All the research protocols involving false gharial were approved by the Institutional Animal Care and Use Committee, Universiti Putra Malaysia, UPM/IACUC/AUP-R020/2015, the Ministry of Natural Resources and Environment, Malaysia (approval number Bil. 5/2015), and the Department of Wildlife and National Park Peninsular Malaysia (approval number B-00477-16-15)**.** Blood samples were obtained from 10 apparently healthy animals provided by Taiping Zoo (Perak) (*n* = 1), National Zoo (Kuala Lumpur) (*n* = 4), and the PERHILITAN Wildlife Conservation Centre (Selangor) (*n* = 5). Animals sampled were classified according to their gender (*n* = 7/10 females: *n* = 3/10 males), and age (*n* = 5/10 adult: *n* = 5/10 juvenile). Sample analysis was performed at the Haematology and Clinical Biochemistry Laboratory, Faculty of Veterinary Medicine, Universiti Putra Malaysia.

#### 2.1.2. False Gharial Restraint

**Figure 1:**
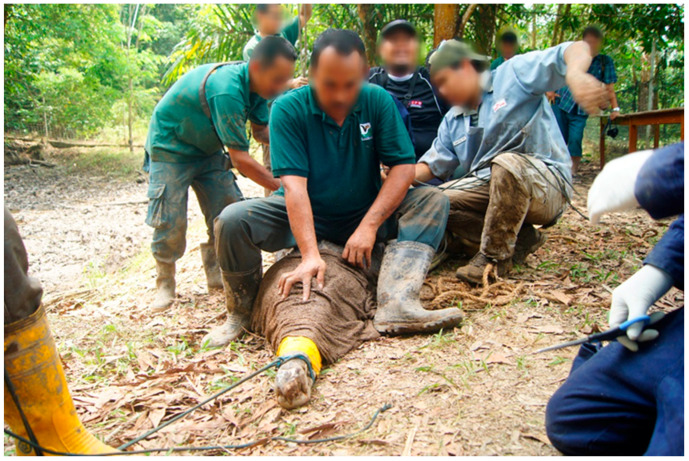
Showing false gharial restraint, where a few people sit on top of the false gharial to pin the animal down for safety.

Using a rope the upper jaw of the false gharial was snared, the snared false gharial was then slowly and carefully pulled to the bank. Before the false gharial could be restrained, they were first blindfolded, by approaching the false gharial from the front and carefully placing a moist sack over the head to cover the eyes. This procedure limits visual stimulation to the false gharial. Lack of visual stimulation reduces movement and struggling, because the animals become disoriented [14]. Care was taken to ensure the nostril was not covered. To pin the head, a few people slowly sat on the neck while pressing the head to keep the jaw closed. The body was also pinned down by other people sitting on the back of the false gharial. This procedure also allows the control of the front and hind limbs. Strong pieces of rope and banding tape were used to secure the jaw. The limbs were also secured by tying them to the body (Figure 1).

#### 2.1.3. False Gharial Physical Examination, Measurement, and Sexing

Immediately after the restraint, all the animals were examined physically, and all the false gharials were found to have a sound locomotive system with no lacerations on their external body. Physical measurements were also carried out, and the morphology of significant parts of the false gharials were measured using a measuring tape by two people and reported in the physical form measurement. Physical measurement was taken of the snout–eyes length (SEL), dorsal–cranial length (DCL), snout–pelvis length (SPL), snout–scute junction (SSJ), tail length (TaL), and total length (TL).

The sex of each false gharial was determined by inserting a gloved finger, into the cloaca and the copulatory organ for palpation and confirmation. The penis of the false gharial is located on the ventral posterior surface of the cloaca near the vent. The male false gharial has a single obvious penis with a fleshy head and cartilaginous shaft, while the female false gharial has a clitoris at a similar location to the male penis. The clitoris is similar in shape to the penis but it is not cartilaginous like the penis and much smaller.

#### 2.1.4. False Gharial Blood Sampling and Transportation

**Figure 2:**
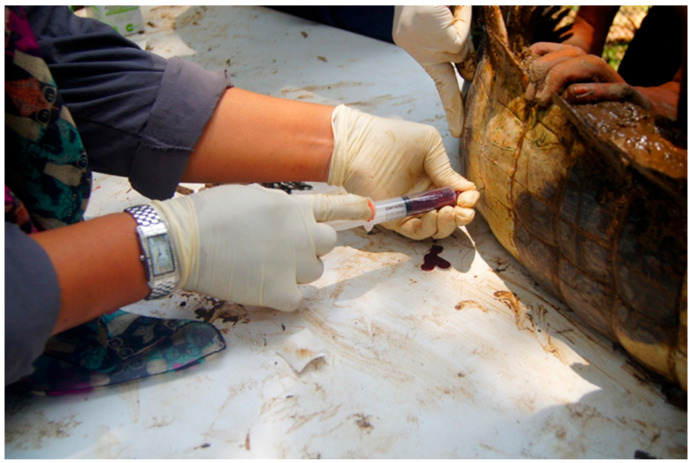
Bleeding procedure for the false gharial, where blood was drawn from the lateral caudal vein using an 18G hypodermic needle.

Blood samples were collected from the lateral caudal vein using an 18G hypodermic needle and/or an 18/20G spinal needle fitted to a 10 mL plastic syringe. The area (Figure 2), was swabbed with 70% alcohol, then 10 mL of blood was drawn aseptically. Each blood sample was divided into a vacutainer without anticoagulant for biochemical analysis, and a lithium heparin vacutainer (containing sodium heparin) (Vacutainer^®^: Becton Dickinson) for haematological studies. Blood in the vacutainer without anticoagulant and the lithium heparin vacutainer were transported within 24 h of collection in a polyethylene container with ice to the Haematology and Clinical Biochemistry Laboratory, Faculty of Veterinary Medicine, Universiti Putra Malaysia.

### 2.2. Hematology

Packed cell volume (PCV) was determined by the standard microhematocrit technique. The blood was mixed thoroughly and drawn into a haematocrit tube of about ¾ lengths. The free end was flame sealed, and subsequently, the haematocrit tubes were centrifuged in a microhematocrit centrifuge (Haematokrit 20; HettichZentrifugen, Tuttlingen, Germany) at 10,000 rpm for 5 min. The PCV was read directly from the microhematocrit reader and recorded. Total erythrocyte counts were obtained by manual counting, using a hemocytometer set (Hawskley B.S.748, London, UK). Leucocyte count was obtained by automatic counting using a Haematology Analyser (CELL-DYN 3700) as described by [15]. A refractometer was used to determine the plasma protein in the haematocrit tube, and the values were expressed as g/L. Blood smears were stained with Wright’s stain (Sigma^®^, St. Louis, MO, USA) to record the morphology of erythrocytes and leukocytes. The differential leukocyte count was obtained from the microscopic evaluation of stained blood smears using a light microscope (Lieca^®^ DME, Wetzlar, Germany) under oil immersion (×100). The battlement counting method was consistently used to obtain representative values from the blood smear.

### 2.3. Blood Morphology and Cells Count

Two fresh blood smears from each sample were prepared for differential leukocyte count and to determine the morphology of blood cells and cell count. The morphology of blood cells was determined by randomly measuring the size, and observing and recording their shape, appearance, and colour. A representative of 10 erythrocytes, 10 heterophils, 10 eosinophils, 10 basophils, 5 lymphocytes, and 5 monocytes were selected from each slide for morphological determinations. The cells were measured using the reticle scale (µm) unit using a light microscope under oil immersion (×100) and analysed using a Nikon^®^ ACT-UP system.

### 2.4. Serum Biochemistry

The biochemical parameters analysed were sodium (Na^+^), potassium (K^+^), chloride (Cl^-^), calcium (Ca_2_^+^), inorganic phosphate, blood urea nitrogen (BUN), creatinine, glucose, cholesterol, bilirubin (total), total protein (TP), globulin, alanine aminotransferase (ALT), alkaline phosphatase (ALP), aspartate aminotransferase (AST), creatine kinase (CK), albumin (Alb), and gamma-glutamyl transferase (GGT). The biochemical analysis was performed using a Chemistry Analyser Automatic (HITACHI 902^®^), as described by [16]. The globulin (Glob) value and the albumin–globulin ratio were calculated using the following formula:

Globulin = TP − Alb

Alb/Glob ratio = Alb/(TP − Alb)

## 3. Results

The results of the present study are presented herein in Table 1, Table 2, Table 3, Table 4, Table 5, Table 6, Table 7 and Table 8, however, statistical analyses were not conducted because the sample size was too small (*n* = 10), as such statistical analysis was not applicable [17].

### 3.1. Erythrocyte Cell and Nucleus Size

The peripheral blood cells of the false gharials showed erythrocyte cells, which are oval in shape and have a centrally located prominent round or oval nucleus. The overall patterns for erythrocyte cell length and width, and also patterns for nuclear length and width, are presented in Table 1. The male false gharial has a greater width than females in terms of both erythrocyte cell and nuclear size.

**Table 1:** Erythrocyte parameters of both the male and female false gharial. For each parameter, the mean (µm) and range (µm) of erythrocyte cells (*n* = 10) and nucleus size are reported.

Parameter	Male (*n* = 3)		Female (*n* = 7)	
	Mean	Range	Mean	Range
Cell length	16.96	15.82–18.33	15.41	14.05–16.84
Cell width	8.47	7.86–9.21	7.14	6.87–7.65
Nuclear length	6.17	5.83–6.76	5.74	5.35–6.12
Nuclear width	3.71	3.46–3.92	3.53	3.14–3.87

### 3.2. Leukocyte Cell

In this study, five types of leukocyte cells were encountered in the peripheral blood of false gharial. The leukocytes consisted of three types of granulocytes, the heterophils, eosinophils, and basophils, and two types of agranulocytes, which included lymphocytes and monocytes. Table 2 shows the mean and range of each leukocyte’s size in captive false gharial.

**Table 2:** Mean (µm) and range (µm) of leukocyte cell measurement in both male and female captive false gharial (*n* = 10).

Parameter	Mean	Range
Heterophils (*n* = 10)	17.5	12.88–20.09
Lymphocytes (*n* = 5)	9.46	7.73–10.14
Monocytes (*n* = 5)	12.27	10.63–15.00
Eosinophils (*n* = 10)	15.28	12.32–18.16
Basophils (N10)	14.52	12.32–16.51

### 3.3. Morphology of Peripheral Blood Cells of Captive False Gharial

**Figure 3:**
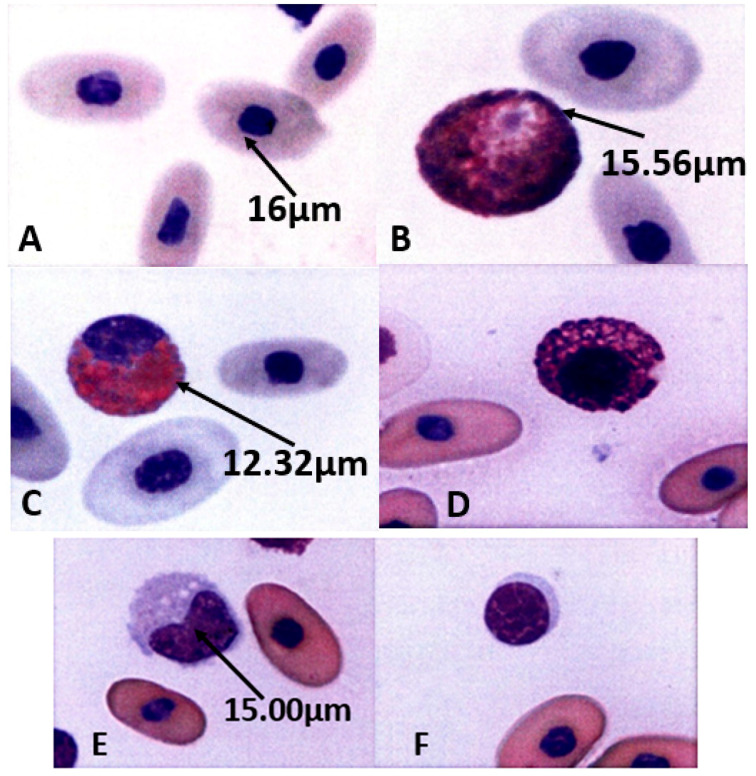
Morphology of peripheral blood cells of captive false gharial. (**A**): Showing erythrocytes of *Tomistoma schlegelii,* observed cells were ellipsoidal and homogenous in color, size, and shape, with centrally positioned oval dense round nuclei. (×100). (**B**): Showing heterophils of *T. schlegelii*, which are the largest and the most common leukocytes in the peripheral blood. Observed cells were large, with a characteristic fusiform acidophilic cytoplasmic granule and a round to ovule shape with an eccentric nucleus. (×100). (**C**): Showing eosinophils of *T. schlegelii*, observed cells were large with a round to spherical eosinophilic cytoplasmic granule. The nuclei are variable in shape, and could be slightly elongated, to lobulated with coarse, clumped chromatin that stains purple. (×100). (**D**): Showing basophils of *T. schlegelii*, observed cells were spherical and filled with basophilic metachromatic granules. The nuclei were slightly eccentric and obscured by the cytoplasmic granules. (×100). (**E**): Showing monocytes of *T. schlegelii*, observed cells had a variable shaped nucleus that varied from oval to irregular, with a finely granular and often vacuolated cytoplasm. (×100). (**F**): Showing lymphocytes of *T. schlegelii*, observed cells had a round or slightly indented nucleus and basophilic cytoplasm. The eccentrically positioned and nuclear chromatin was heavily clumped. (×100).

Morphology of Peripheral Blood Cells of Captive False Gharial (Figure 3).

### 3.4. Hematological and Serum Biochemical Values Determined from Captive False Gharial

Please check Table 3, Table 4, Table 5, Table 6, Table 7 and Table 8.

**Table 3:** Hematological Profile of Male and Female False Gharial (*n* = 10). For each parameter, the mean and the standard deviation (SD), as well as the minimum (Min) and maximum (Max) range are reported.

	Parameter	Unit	Mean	SD	Min–Max
1	PCV	%	18.90	4.70	19–24
2	Erythrocytes	×10^12^/L	0.46	0.17	0.180–0.657
3	Haemoglobin	g/L	76.36	52.56	28.70–112.00
4	MCV	fL	452.66	156.34	337–870
5	MCHC	g/L	398.38	67.11	246–467
6	Leukocytes	×10^9^/L	40.45	44.32	3–104
7	Heterophils	%	56.90	10.31	40–77
8	Heterophils	×10^9^/L	22.99	25.67	1.98–61.61
9	Lymphocytes	%	17.60	8.77	2–31
10	Lymphocytes	×10^9^/L	7.10	7.70	0.06–18.72
11	Monocytes	%	10.60	6.21	2–20
12	Monocytes	×10^9^/L	4.28	6.68	0.0–20.4
13	Eosinophils	%	9.70	7.21	1–27
14	Eosinophils	×10^9^/L	3.92	5.28	0.48–14.56
15	Basophils	%	5.40	5.15	2–10
16	Basophils	×10^9^/L	2.18	2.08	0.21–6.12

Note: PCV = parked cell volume, MCV = mean corpuscular volume, MCHC = mean corpuscular haemoglobin concentration.

**Table 4:** Serum Biochemical Values of Male and Female False Gharial (*n* = 10). For each parameter the mean and the standard deviation (SD), as well as the minimum (Min) and maximum (Max) range are reported.

	Parameter	Unit	Mean	SD	Min–Max
1	Total protein	g/L	50.24	5.19	43.4–59.3
2	Albumin	g/L	10.26	1.63	7.4–13.0
3	Globulin	g/L	39.98	4.32	34.3–47.4
4	Alb: Glob ratio	-	0.26	0.04	0.20–0.34
5	Calcium	mmol/L	3.08	1.28	1.86–6.57
6	Cholesterol	mmol/L	4.51	1.24	2.05–6.19
7	Glucose	mmol/L	3.84	1.70	1.8–7.7
8	Phosphorus	mmol/L	1.81	0.39	1.20–2.27
9	Triglyceride	mmol/L	2.29	4.06	0.39–13.66
10	Sodium	mmol/L	143.56	16.77	104.6–160.1
11	Potassium	mmol/L	4.45	0.87	3.3–6.0
12	Chloride	mmol/L	100.94	18.16	68.8–121.0
13	Urea nitrogen	mmol/L	0.48	0.32	0–0.9
14	Uric acid	µmol/L	194.08	92.08	114.6–366.9
15	Creatinine	µmol/L	32.00	3.02	28.0–35.0
16	Bilirubin	µmol/L	5.73	1.57	3.6–8.6
17	Creatine kinase	U/L	2882.40	1538.34	1085–6190
18	ALT	U/L	16.97	6.07	5.7–27.5
19	AST	U/L	41.35	23.77	18.5–82.1
20	ALP	U/L	18.58	3.00	12.9–22.6

Note: ALT = alanine aminotransferase, AST = aspartate transaminase, ALP = alkaline phosphatase.

**Table 5:** Comparison of the Hematological Profile between Male and Female False Gharial (*n* = 10). For each parameter the mean and the standard deviation (SD), as well as the minimum (Min) and maximum (Max) range are reported.

	Parameter	Unit	Males (*n* = 3)			Females (*n* = 7)		
			Mean	SD	Min–Max	Mean	SD	Min–Max
1	PCV	%	20.20	0.45	20–21	17.60	6.73	19–24
2	Erythrocytes	×10^12^/L	0.53	0.06	0.430–0.593	0.38	0.22	0.180–0.657
3	Haemoglobin	g/L	86.12	5.75	78.5–93.9	66.60	34.63	28.7–112.0
4	MCV	fL	385.27	48.80	337–465	520.04	203.12	3530–870
5	MCHC	g/L	426.20	22.19	393–447	370.56	87.79	246–467
6	Leukocytes	×10^9^/L	28.72	40.90	7–101	52.18	49.01	3–104
7	Heterophils	%	58.00	14.70	47–77	57.80	4.82	54–66
8	Heterophils	×10^9^/L	16.73	25.17	2.82–61.61	29.26	27.39	1.98–59.16
9	Lymphocytes	%	14.20	10.28	6–31	14.00	8.22	2–18
10	Lymphocytes	×10^9^/L	5.09	7.26	0.42–17.17	9.10	8.40	0.06–18.72
11	Monocytes	%	4.40	3.91	1–10	7.20	8.17	0–20
12	Monocytes	×10^9^/L	1.94	3.00	0.12–7.07	6.62	8.81	0–20.4
13	Eosinophils	%	16.80	5.93	13–27	10.60	7.60	1–18
14	Eosinophils	×10^9^/L	3.98	5.16	0.98–13.13	3.85	6.01	0.48–14.56
15	Basophils	%	6.80	5.17	2–15	10.20	5.07	4–15
16	Basophils	×10^9^/L	1.01	0.69	0.21–2.02	3.35	2.43	0.45–6.12

**Table 6:** Comparison of the Biochemical Values between Male and Female False Gharial (*n* = 10). For each parameter the mean and the standard deviation (SD), as well as the minimum (Min) and maximum (Max) range are reported.

	Parameter	Unit	Males (*n* = 3)			Females (*n* = 7)		
			Mean	SD	Min–Max	Mean	SD	Min–Max
1	Total protein	g/L	47.12	2.28	43.40–49.40	53.36	5.58	44.80–59.30
2	Albumin	g/L	10.22	1.36	9.10–12.60	10.30	2.02	7.40–13.00
3	Globulin	g/L	36.90	1.60	34.3–38.6	43.06	3.98	37.4–47.4
4	Alb:Glb ratio	-	0.28	0.04	0.25–0.34	0.24	0.03	0.21–0.28
5	Calcium	mmol/L	2.53	0.43	1.86–2.85	3.63	1.66	2.68–6.57
6	Cholesterol	mmol/L	3.77	1.06	2.05–4.91	5.24	1.01	3.76–6.19
7	Glucose	mmol/L	171.14	1874.62	90.6–343.8	145.92	1295.78	60.2–220.9
8	Phosphorus	mmol/L	3.44	0.75	2.5–4.5	4.24	2.36	1.8–7.7
9	Triglyceride	mmol/L	1.81	0.50	1.42–2.34	1.82	0.30	1.47–2.17
10	Sodium	mmol/L	1.00	0.95	0.53–2.71	3.58	5.66	0.39–13.66
11	Potassium	mmol/L	137.98	21.91	104.6–154.9	149.14	8.65	139.9–160.1
12	Chloride	mmol/L	4.40	1.12	3.3–6.0	4.50	0.64	3.8–5.5
13	Urea nitrogen	mmol/L	99.72	23.67	68.8–118.4	102.16	13.33	87.2–121.0
14	Uric acid	µmol/L	116.88	111.83	114.6–366.9	221.28	68.72	154.3–324.1
15	Creatinine	µmol/L	30.00	2.35	28.0–33.0	34.00	2.24	31.0–37.0
16	Bilirubin	µmol/L	0.74	0.11	0.60–0.90	0.22	0.20	0–0.4
17	Creatine kinase	U/L	6.34	0.67	5.3–7.1	5.12	2.05	3.6–8.6
18	ALT	U/L	14.60	6.57	5.7–20.7	19.34	5.06	14.4–27.5
19	AST	U/L	38.96	26.90	18.5–82.1	43.74	23.09	24.6–81.3
20	ALP	U/L	19.84	3.94	12.90–22.60	17.32	0.85	16.30–18.10

**Table 7:** Comparison of the Hematological Profile between Two Environments. For each parameter the mean and the standard deviation (SD), as well as the minimum (Min) and maximum (Max) range are reported.

	Parameter	Unit	Zoo (*n* = 5)			Sg Dusun (*n* = 5)		
			Mean	SD	Min–Max	Mean	SD	Min–Max
1	PCV	%	16.40	5.50	19–24	21.40	1.95	20–24
2	Erythrocytes	×10^12^/L	0.39	0.17	0.18–0.56	0.52	0.17	0.23–0.657
3	Haemoglobin	g/L	69.52	28.71	28.70–93.90	83.20	23.00	49.10–93.30
4	MCV	fL	434.40	59.16	357–500	470.91	225.08	337–870
5	MCHC	g/L	411.60	53.03	319–447	385.16	82.98	246–467
6	Leukocytes	×10^9^/L	6.40	2.07	3–8	74.50	38.93	21.6–104.0
7	Heterophils	%	62.00	11.45	47–77	53.80	8.14	40–61
8	Heterophils	×10^9^/L	3.96	1.50	1.98–5.39	42.03	23.96	8.64–61.61
9	Lymphocytes	%	7.20	3.42	2–11	21.00	6.52	17–31
10	Lymphocytes	×10^9^/L	0.51	0.30	0.06–0.88	13.68	4.97	6.70–18.72
11	Monocytes	%	1.20	0.84	0–2	10.40	5.77	5–20
12	Monocytes	×10^9^/L	0.11	0.07	0–0.17	8.45	7.54	2.16–20.40
13	Eosinophils	%	18.40	5.03	14–27	9.0	6.04	1–14
14	Eosinophils	×10^9^/L	1.18	0.45	0.48–1.62	6.65	6.61	1.02–14.56
15	Basophils	%	11.20	5.50	3–15	5.80	3.35	2–11
16	Basophils	×10^9^/L	0.68	0.39	0.21–1.20	3.69	2.00	0.50–2.08

**Table 8:** Comparison of biochemical values, showing the parameters Mean, SD, and Range, between male and female captive false gharials in Peninsular Malaysia.

	Parameter	Unit	Zoo (*n* = 5)			Sg Dusun (*n* = 5)		
			Mean	SD	Min–Max	Mean	SD	Min–Max
1	Total protein	g/L	2.53	0.43	1.86–2.85	3.63	1.66	2.68–6.57
2	Albumin	g/L	14.60	6.57	5.7–20.7	19.34	5.06	14.4–27.5
3	Globulin	g/L	38.96	26.90	18.5–82.1	43.74	23.09	24.6–81.3
4	Alb: Glb ratio	-	3.77	1.06	2.05–4.91	5.24	1.01	3.76–6.19
5	Calcium	mmol/L	3111.60	1874.62	1632–6190	2653	1295.78	1085–3977
6	Cholesterol	mmol/L	3.44	0.75	2.5–4.5	4.24	2.36	1.8–7.7
7	Glucose	mmol/L	1.81	0.50	1.42–2.34	1.82	0.30	1.47–2.17
8	Phosphorus	mmol/L	1.00	0.95	0.53–2.71	3.58	5.66	0.39–13.66
9	Triglyceride	mmol/L	137.98	21.91	104.6–154.9	149.14	8.65	139.9–160.1
10	Sodium	mmol/L	4.40	1.12	3.3–6.0	4.50	0.64	3.8–5.5
11	Potassium	mmol/L	99.72	23.67	68.8–118.4	102.16	13.33	87.2–121.0
12	Chloride	mmol/L	116.88	111.83	114.6–366.9	221.28	68.72	154.3–324.1
13	Urea nitrogen	mmol/L	0.74	0.11	0.60–0.90	0.22	0.20	0–0.4
14	Uric acid	µmol/L	30.00	2.35	28.0–33.0	34.00	2.24	31.0–37.0
15	Creatinine	µmol/L	6.34	0.67	5.3–7.1	5.12	2.05	3.6–8.6
16	Bilirubin	µmol/L	19.84	3.94	12.90–22.60	17.32	0.85	16.30–18.10
17	Creatine kinase	U/L	47.12	2.28	43.40–49.40	53.36	5.58	44.80–59.30
18	ALT	U/L	10.22	1.36	9.10–12.60	10.30	2.02	7.40–13.00
19	AST	U/L	36.90	1.60	34.3–38.6	43.06	3.98	37.4–47.4
20	ALP	U/L	0.28	0.04	0.25–0.34	0.24	0.03	0.21–0.28

## 4. Discussion

Our present study employed a direct sampling method, taking into consideration the selection criteria (biological, clinical, and geographic) such as age, sex, history, location, and environment [17], and was conducted on a small sample size, because of the vulnerable conservation status of the animal [7]. Therefore, in the results of the studies presented above, statistical analyses were not conducted because the sample size was too small (*n* = 10), and, as such, statistical analysis was not applicable [17].

Even though the blood cells of all crocodilian species are nucleated, with oval erythrocytes containing round to oval nuclei, and which are centrally located, in this study we reported differences in the erythrocyte morphology of false gharial, where it was observed that false gharials have smaller erythrocyte dimensions of 15.9 µm length and 7.6 µm width, compared to 23.0 µm length and 14.3 µm width in *Alligator mississippiensis* [18], and *Caiman Crocodilus,* which has 17.0 µm length and 9.0 µm width [19].

Moreover, in this study, the obtained PCV values of the false gharials ranged between 19–24%, and after the values were compared between males and females, the obtained mean value in males was still higher than in females. In general, reptiles have lower hematocrits, of around 20–35% [20], and a PCV <18–20% is indicative of anemia in a reptile individual [21].

Hence, our obtained values (Mean value of PCV) are consistent with the reported normal range of reptiles [22] and do not indicate evidence of anemia in a reptile, because the obtained value was not lower than 18%. Similarly, in a related study, [23] reported the PCV values of 17–28%, with a mean of 23.36 in a *Caiman Crocodilus fuscus.* As such, the values obtained in our study do not indicate anemia, evident by the normal values of MCV and MCHC.

It is important to note that, the result of our present study was contrary to the previous values reported by [13]; where in our study the PCV values were 18.90%, while that of [13] was 15.2%, the hemoglobin in our study was 76.4 g/L, while in a similar study by [13], 71.0 g/L was recorded. The erythrocyte values of our study were 0.46 × 10^12^/L, while the previous study reported 0.34 × 10^12^/L. These discrepancies could be due to the stress associated with either capture and or handling during restraint, and thereby affecting the hematologic and biochemical values [24].

The total leukocytes in our study (40.45 × 10^9^/L) were higher than the previous study (4.35 × 10^9^/L) in false gharial by [13]; this could be due to stress [25] from the handling process or physical restraint of the false gharials in the study, or could be due to seasonal fluctuation [26]. Similarly, the heterophils values in our study (57.90%) were higher than the previous study (64.9%) of false gharial by [13], and the lymphocytes values of our study (14.10%) were lower than the previous study (24.1%) in false gharial [13], these lymphopenia and heterophillia values could be due to stress responses [22].

The eosinophils and monocytes values of this study (13.70% and 5.80%) were higher than the previous study (8.5% and 3.9%) in false gharial [13]. However, the previous study does not report the values of basophils, which our study reported (8.50%). These discrepancies between the values of the previous study and the values obtained in our study could be due to factors such as age, sex, ambient temperature and season, environment, diet, and species, as well as the method that was used [27,28].

However, the results of our study agreed with reported findings that the hematology and serum biochemistry values of reptiles vary with season, age, sex, and serial sampling, as well as between various laboratories, where reported normal values differ [29], which is evident from our obtained values and the comparison.

In terms of sex, our present study indicates that female false gharial had a lower mean in erythrocytes (0.38 × 10^12^/L) and hemoglobin (66.60 g/L) than males (0.53 × 10^12^/L and 86.12 g/L). While the leukocytes mean was lower in males (28.72 × 10^9^/L) than in females (52.18 × 10^9^/L), these values agreed with the findings that gender and size affect the hematological and serum biochemical values [26]. This is supported by the composition of the false gharials in our study (*n* = 5 adult *n* = 5 juvenile).

Furthermore, our study revealed that the hematological values, showing the number of erythrocytes (0.52 × 10^12^/L), leukocytes (74.50 × 10^12^/L), and hemoglobin (83.20 g/L), in false gharials kept in PERHILITAN Wildlife Conservation Centre with a semi-wild environment, had a higher mean than the false gharials kept in Zoos (0.39 × 10^12^/L, 6.40 × 10^12^/L, and 69.52 g/L) on a concrete pond.

This is assumed to be because the captive pond at PERHILITAN Wildlife Conservation Centre has a mud floor, so the disturbance of the bottom substrate created high turbidity of water in the pond, and as such the high content of suspended material in the water might bring about possible pathogenic organisms, which probably caused the physiological functions of the false gharials to work harder in fighting water-borne pathogens.

For other crocodilian species, the results of hematological values obtained from this study were contrary to the hematological values of saltwater crocodile (*Crocodylus porosus*) as reported by [30]. The total leukocytes count in our present study (3–104 × 10^9^/L) was considerably higher than in the salt-water crocodile (6.4−25.7 × 10^9^/L) as reported by [30]. Except for the ranges for lymphocytes, all other leucocyte counts showed considerable differences between the false gharial and the salt-water crocodile values. In general, the values in our study differed from the normal ranges of other reptiles, as reported by [22].

The lymphocyte count for salt-water crocodile (4.5–21.6 × 10^9^/L) was considerably greater than that of false gharial in our study (0.06–18.72 × 10^9^/L), these could be due to the effect of the season at the time of the study, as lymphocytes were reported to be lower in the animal during ecdysis and winter, than summer months [31]. However, the higher leucocyte count for the false gharial was most likely influenced by the condition of captivity, as well as the sample size [32], because in a captive habitat it is the alternative pond that replaces the natural freshwater, and probably the pathogens in the water differ, which might cause pollution of the water, thereby stimulating the immune system of the captive false gharial to work harder as a defense mechanism.

The natural habitat of the false gharial is the peat swamp forest, which is threatened by habitat destruction due to land transformation, agricultural activity, illegal logging, and forest fires, resulting in habitat pollution and changes in the flora of freshwater pathogens [1,3,33].

However, the result of serum biochemistry values obtained in our study, revealed that the means of total protein (50.24 g/L), calcium (3.08 mmol/L), cholesterol (4.51 mmol/L), triglycerides (2.29 mmol/L), creatinine (32.00 µmol/L), and ALP (18.58 U/L) were considerably higher than that of the previous study (37.0 g/L, 2.55 mmol/L, 2.86 mmol/L, 0.52 mmol/L, 18.56 µmol/L and 17.8 U/L) on false gharial by [13], while the obtained values of phosphorus, potassium, and uric acid were inconsistent, while no values were recorded by [13] for parameters like albumin, globulin, creatine kinase, AST, and ALT.

The glucose value obtained in our study (3.84 mmol/L) is within the normal range and agrees with the findings of [22], who reported the normal glucose values of reptiles to be 60 mg/dL–100 mg/dL (3.3 mmol/L–5.6 mmol/L). Generally, glucose level varies among reptiles due to factors such as environment, nutrition, and sampling time [34]. However, a high level of glucose was reported in individual reptiles whose blood was collected immediately after feeding, and likewise a low level of glucose was reported in a crocodile subjected to capture stress [34].

Uric acid is reported to be the main excretory waste product in the urine and feces of reptiles [27], however, our obtained mean values of 194.08 µmol/L (2.19 mg/dL) were within the normal range of 0 mg/dL–10 mg/dL reported by [22] and agreed with the reported range findings of [24], while they were contrary to the findings of 3.3 mg/dL by [30]. These could be due to factors such as diet, time of feeding, and the corresponding time of blood sampling and processing, as high values have been reported a day after an individual reptile had eaten [22].

For urea and creatinine values, it is important to note that reptiles are known as uricotelic, and, depending on their hydration status, produce large quantities of urea, hence values for urea and creatinine are unreliable indicators of reptile clinical pathology and not considered important in assessing renal disease in reptiles [22,35].

ALT and AST activity was reported to be increased during liver diseases, and as such play a role in clinical diagnosis [35], where a value less than 20 IU/L was considered normal in reptiles [27].

We also compared the values obtained in our study between the sexes, where the mean of total protein (53.36 g/L), globulin (43.06 g/L), calcium (3.63 mmol/L), cholesterol (5.24 mmol/L), phosphorus (4.24 mmol/L), urea nitrogen (102.16 mmol/L), uric acid (221.28 µmol/L), creatinine (34.00 µmol/L), ALT (19.34 U/L), and AST (43.74 U/L) in female false gharials were higher than in males (47.12 g/L, 36.90 g/L, 2.53 mmol/L, 3.77 mmol/L 3.44 mmol/L, 99.72 mmol/L 116.88 µmol/L 30.00 µmol/L 14.60 U/L 38.96 U/L). This could be due to the effect of sex and gender [26]; at the same time an increase in creatinine kinase and AST values in wild mammals was reported [24,25,36] to be associated with muscle or soft tissue injury following excitement and stress during capture events.

However, the albumin–globulin (Alb: Glob) ratio, glucose, bilirubin, creatine kinase, and ALP were higher in males than in females, and this could be due to the effect of gender and size, which are known to affect hematological and biochemical values [26], while in the obtained values for albumin, triglyceride, and chloride there were no differences between sexes.

The differences in biochemistry values between false gharial in different locations, total protein, albumin, globulin, and Alb: Glob ratio were higher in false gharials that were kept in the PERHILITAN Wildlife Conservation Centre than false gharials in zoos. This is probably based on the diet management of the PERHILITAN Wildlife Conservation Centre, where the animals are given extra feed, like catfish, once a month. This suggests that the diet and other physiological conditions of the animals can influence the biochemistry values.

Hence, our present study provides more information compared to the similar previous study, because it furthers the knowledge of the hematology and serum biochemistry of the false gharial by reporting the complete blood parameter values, which are expected to offer a guide toward understanding the pathology and pathogenesis of diseases affecting the false gharial [21].

## 5. Conclusions and Recommendation

In conclusion, the results obtained in this study indicate that false gharial has a smaller erythrocytes dimension compared to other crocodilian species. False gharials in natural captive conditions show more leukocytes than those kept in zoos, indicating that habitat and environmental factors may affect hematological parameters. Moreover, biochemistry values also show differences between false gharials in different environmental conditions. The authors are aware of the small sample size, nevertheless, few data are available concerning the blood parameters in false gharial, or on their health status. Therefore, the data obtained by this study could provide a baseline for further research, as well as being a useful tool for clinical diagnosis.

## Data Availability

Data sharing is not applicable to this article.
